# Editorial: Insights in hyperprolactinemia

**DOI:** 10.3389/fendo.2023.1351471

**Published:** 2024-01-11

**Authors:** Maria M. Pineyro, Susana B. Rulli, Gianluca Tamagno

**Affiliations:** ^1^ Clínica de Endocrinología y Metabolismo, Hospital de Clínicas, Facultad de Medicina, Universidad de la República, Montevideo, Uruguay; ^2^ Centro de Estudios Biomédicos Básicos, Aplicados y Desarrollo (CEBBAD) Universidad Maimónides, Buenos Aires, Argentina; ^3^ Department of Medicine, Blackrock Clinic and Hermitage Clinic - Blackrock Health, Dublin, Ireland

**Keywords:** hyperprolactinemia, AIP (aryl hydrocarbon receptor interacting protein), quinagolide, prolactin (PRL), PCOS (polycystic ovarian syndrome)

Hyperprolactinemia is the most common endocrine disorder of the hypothalamic-pituitary axis. Prolactin is synthesized by the anterior pituitary lactotroph cells and is regulated by various factors, with dopamine being the primary inhibitory factor. There are multiple etiologies of hyperprolactinemia and an accurate diagnosis is crucial for appropriate treatment (1).

This Research Topic, presented as a Research Topic issue of the journal, offers valuable insights into the multifaceted nature of hyperprolactinemia. These articles, that cover aspects ranging from studies on herbal remedies to genetic links, diagnostic criteria, and diagnostic or therapeutic tools, aim to contribute to a more comprehensive understanding of this intricate endocrine disorder.

Rodier et al. have explored the innovative use of metoclopramide test in the context of hyperprolactinemia among women with polycystic ovarian syndrome (PCOS). This study repurposes an existing diagnostic tool to assess its efficacy in a different clinical context, potentially streamlining the diagnostic process and enhancing our understanding of hyperprolactinemia in a specific patient population such as women with PCOS. Despite the limitations represented by the retrospective nature of the study and by the fact that most patients had normal prolactin on a second check, this novel approach could offer an efficient and cost-effective diagnostic method, presenting implications for improved management and understanding of hyperprolactinemia within the context of PCOS.

One of the most challenging aspects for the successful management of hyperprolactinemia is represented by the correct diagnosis of the underlying etiology. Cho et al. have studied the issue of the correct distinction between prolactinomas and other small sellar lesions. With that regard, they have evaluated the use of the prolactin-volume-ratio as a diagnostic and discriminatory tool. In patients with small sellar lesions, the prolactin-volume-ratio could serve as a potential predictive indicator for diagnosing prolactin-producing pituitary adenomas. By refining the diagnostic criteria, this research may contribute to the optimization of patient care, recognizing that different clinical conditions may require distinct approaches. Limitations include de retrospective nature of the study, as well as being a single center analysis with a limited number of patients. This study may serve also as an incentive to further assess the prolactin-volume-ratio in small tumors within the sellar region.

An article by Zeng et al. covers the effectiveness and safety of quinagolide, a dopamine agonist used to treat hyperprolactinemia. This systematic review and meta-analysis provides valuable insights into the current state of the pharmacological management of this condition. Though cabergoline and bromocriptine are the most widely used dopamine agonists for the treatment of hyperprolactinemia and the majority of the studies about the use of quinagolide in such a setting are quite old, this review helps and informs the healthcare providers about the role of quinagolide, a non-ergot-like dopamine agonist, in the treatment of hyperprolactinemia and may contribute to empowering them to make an informed decision about the treatment options and the related safety concerns.

Vroonen et al. have explored the genetic aspects of hyperprolactinemia, specifically focusing on the link between germline pathogenic variants of the aryl hydrocarbon receptor interacting protein (AIP) gene and prolactinoma characteristics. This study reveals unique characteristics such as aggressive clinical features and larger or invasive tumors at young age in patients with an AIP germline variant compared to controls. These findings emphasize the significance of genetic factors in influencing prolactinoma development. Limitations include its retrospective design and the fact that it is a single-center analysis with a restricted number of participants. By delving into the genetic factors that may influence this condition, the authors promote a more precise and personalized approach to its management.

Finally, the work of Puglia et al. on Vitex agnus castus (VAC), a well-known herbal remedy, explores the effects of this phytotherapic drug on hyperprolactinemia by reviewing the relevant literature. It is hypothesized that the diterpenes found in VAC extracts may be able to engage with dopamine D2 receptors and lead to the inhibition of prolactin release through the activation of these receptors in the anterior pituitary. While the conventional treatment of hyperprolactinemia includes dopamine agonists to suppress prolactin secretion, this paper investigates the potential use of an alternative and complementary therapy for the management of hyperprolactinemia in selected patients. This review article may open new avenues for the treatment of some patients with hyperprolactnemia who are seeking a natural medication for the treatment of their condition. However, to ensure the effectiveness and safety of VAC, more research on its physiology and pharmacological mechanism of action is required.

In conclusion, this Research Topic provides a multifaceted exploration of hyperprolactinemia, offering valuable insights that span from etiological to diagnostic and therapeutic aspects of this fascinating topic. From the impact of genetics to the application of either established or innovative diagnostic tools and to the role of conventional and alternative therapeutic options, this Research Topic aims to contribute to a better understanding of the clinical issue represented by hyperprolactinemia, hopefully including relevant aspects in support of evidence-based and precision medicine.

Considering the high frequency by which the endocrinologists face hyperprolactinemia and the diagnostic or therapeutic challenges related to such clinical finding, we believe that every valuable effort for improving and making more solid the scientific approach to this condition is welcome and useful. However, a number of relatively rare challenging or peculiar clinical situations, like the management of aggressive or malignant prolactinomas (2) or the approach to hyperprolactinemia in the elderly or in transgender patients (3), for example, still require further steps towards the optimization of the care for each single individual.

Our understanding of hyperprolactinemia is evolving and the diverse insights presented in the articles of this Research Topic may contribute to a more comprehensive and nuanced perspective on the topic. The implications extend beyond etiology and diagnosis and reach into the realms of personalized treatment, improved clinical profiles, and innovative applications of existing diagnostic tools. Together, these articles pave the way for a more informed and refined approach to hyperprolactinemia, bringing physicians and scientists closer to an optimized and personalized patient care and to an enhanced insight into this intricate endocrine disorder.

## Author contributions

MP: Writing – original draft, Writing – review & editing. SR: Writing – original draft, Writing – review & editing. GT: Writing – original draft, Writing – review & editing.
